# Effectiveness of screening for atrial fibrillation and its determinants. A meta-analysis

**DOI:** 10.1371/journal.pone.0213198

**Published:** 2019-03-20

**Authors:** Pawel Petryszyn, Piotr Niewinski, Aleksandra Staniak, Patryk Piotrowski, Anna Well, Michal Well, Izabela Jeskowiak, Gregory Lip, Piotr Ponikowski

**Affiliations:** 1 Department of Clinical Pharmacology, Wroclaw Medical University, Wroclaw, Poland; 2 Department of Heart Diseases, Wroclaw Medical University, Wroclaw, Poland; 3 Department of Psychiatry, Wroclaw Medical University, Wroclaw, Poland; 4 Institute of Cardiovascular Sciences, University of Birmingham, Birmingham, United Kingdom; University of Mississippi Medical Center, UNITED STATES

## Abstract

**Background:**

Many atrial fibrillation patients eligible for oral anticoagulants are unaware of the presence of AF, and improved detection is necessary to facilitate thromboprophylaxis against stroke.

**Objective:**

To assess the effectiveness of screening for AF compared to no screening and to compare efficacy outcomes of different screening strategies.

**Materials and methods:**

Cochrane Central Register of Controlled Trials, EMBASE and MEDLINE from Jan 1, 2000 –Dec 31, 2015 were searched. Studies employing systematic or opportunistic screening and using ECG or pulse palpation in populations age ≥40 years were included. Data describing study and patient characteristics and number of patients with new AF were extracted. The outcome was the incidence of previously undiagnosed AF.

**Results:**

We identified 25 unique (3 RCTs and 22 observational) studies (n = 88 786) from 14 countries. The incidence of newly detected AF due to screening was 1.5% (95% CI 1.1 to 1.8%). Systematic screening was more effective than opportunistic: 1.8% (95% CI 1.4 to 2.3%) vs. 1.1% (95% CI 0.6 to 1.6%), p<0.05, GP-led screening than community based: 1.9% (95% CI 1.4 to 2.4%) vs. 1.1% (95% CI 0.7 to 1.6%), p<0.05, and repeated heart rhythm measurements than isolated assessments of rhythm: 2.1% (95% CI 1.5–2.8) vs. 1.2% (95% CI 0.8–1.6), p<0.05. Only heart rhythm measurement frequency had statistical significance in a multivariate meta-regression model (p<0.05).

**Conclusions:**

Active screening for AF, whether systematic or opportunistic, is effective beginning from 40 years of age. The organisation of screening process may be more important than technical solutions used for heart rhythm assessment.

## Introduction

Atrial fibrillation (AF) is the most common sustained arrhythmia with the prevalence estimated at 2% of the total adult population[1]. Over the last two decades the prevalence of reported AF has increased by 13% and epidemiological studies predict further increases related mostly to the ageing of the society[2]. AF carries a substantial risk of thromboembolism, heart failure and mortality[3]. These risks of complications related to AF can be substantially decreased by introduction of appropriate treatment. This has been repeatedly demonstrated for oral anticoagulants (OACs) and acute ischemic stroke[4,5]. The nonvitamin-K antagonist OACs may further improve outcomes and decrease the risk of intracranial bleeding[6]. Due to lack of symptoms a significant proportion of patients suffering from AF are unaware of their arrhythmia[7].

Screening for AF aimed at early detection of asymptomatic individuals appears to be soimportant as it enables the implementation of an early intervention and changes the prognosis. According to the current ESC guidelines opportunistic screening for AF is recommended in patients >65 years with the use of pulse-taking or an electrocardiogram (ECG)[8]. The STROKESTOP study findings suggest that systematic screening based on repeated short ECG recordings results in satisfactory yield for AF detection in elderly individuals[9].

It is currently unclear which of these two screening methods is more effective in detecting previously undiagnosed AF. Other factors might also influence the efficiency of the screening process. First, different technical modalities may be employed for the screening, including standard 12-lead ECG, various portable devices recording data other than 12-lead ECG and pulse palpation. Second, organisation of the screening process may vary (GP-led vs. community-based approach).

In this systematic review and meta-analysis we aimed to: (1) assess the effectiveness of active screening for previously undiagnosed AF and (2) investigate various aspects of the screening programme.

## Methods

The study followed the Preferred Reporting Items for Systematic Reviews and Meta-Analyses (PRISMA) flow diagram and checklist (S1 Table) [10]. Details of the protocol for this systematic review were registered on PROSPERO and can be accessed at *https*:*//www*.*crd*.*york*.*ac*.*uk/PROSPERO/display_record*.*asp*?*ID=CRD42017067507*

### Literature search

Relevant studies were identified by searching multiple databases including Cochrane Central Register of Controlled Trials, EMBASE and MEDLINE (PubMed). Keyword search terms were ‘atrial fibrillation’ AND (‘mass screening’ OR ‘screening’ OR ‘detection’ OR ‘case finding’) AND (‘pulse’ OR ‘electrocardiography’ OR ‘ecg’ OR ‘electrocardiogram’). Embase database was searched as follows: #1 'atrial fibrillation [definition] OR 'atrial fibrillation [Title/Abstract], #2 'mass screening [definition] OR 'mass screening' [Title/Abstract] OR 'screening' [Title/Abstract] OR 'detection' [Title/Abstract] OR 'case finding' [Title/Abstract], #3 'pulse rate' [Definition] OR 'pulse rate' [Title/Abstract] OR 'electrocardiogram' [Definition] OR 'electrocardiogram' [Title/Abstract] OR 'electrocardiography' [Title/Abstract] OR 'ecg' [Title/Abstract], #4 ‘#1 AND #2 AND #3’. Search terms for MEDLINE and EMBASE with corresponding publication numbers can be found in the S1 Appendix. The search was constrained to the period January 1, 2000 –Dec 31, 2015. No language or other limitations were applied. Reference lists of all included papers were searched to identify potentially relevant articles, so the Internet handsearching was performed.

### Criteria for considering studies

All randomised controlled trials (RCTs) and observational studies comparing screening for AF to no screening were eligible for inclusion. Studies with historical control and in which there was only an intervention arm were also qualified. Case series and review articles were excluded.

Eligible participants were men and women ≥ 40 years living in community or attending GP practices. People with implanted pacemaker or defibrillator likewise any specific groups like athletes were excluded. Patients with previous stroke or TIA were considered only if they constituted a proportion of the larger population. Patients with the prior diagnosis of AF were excluded from the final number of newly diagnosed AF cases.

Studies eligible for inclusion compared systematic or opportunistic screening programmes to no screening in the control or pre-intervention group. “No screening” was defined as a passive approach towards the diagnosis of AF. The latter indicating that the diagnosis was made either incidentally or following presentation with symptoms of arrhythmia over the study period. “Systematic screening” was defined as screening carried out in all people over a certain age or in a particular sub-group. “Opportunistic screening” was defined as screening performed in patients attending medical professional for another reason. The term “active screening” encompasses both systematic and opportunistic screening and means searching for AF in opposition to passive case-finding in people with symptoms or signs of AF referred to here as “no screening”. Screening might have been led by primary care physicians in their local practices (using their facilities) or taken place in community, i.e. in the form of health program, in which different medical centres participated. A detection method of AF in the intervention group could consist of pulse palpation, ECG (less than 12-lead and 12-lead) and the use of some other wearable devices. The heart rate could be checked only once or repeatedly, and the programme could be designed as a one- or multi-step process. The diagnosis of AF needed to eventually be confirmed using 12-lead ECG interpreted by an appropriately trained GP or a cardiologist.

The primary outcome was the incidence of previously undiagnosed AF as the result of screening or the difference in the incidence between the intervention and control (pre-intervention) group. The secondary outcomes comprised the identification of factors influencing the effectiveness of screening, as well as the eligibility of newly detected AF for stroke thromboprophylaxis (assessed with the use of CHADS_2_ or CHA_2_DS_2_-VASc scales).

### Data collection and analysis

Preliminary screening of retrieved abstracts in order to eliminate irrelevant studies was performed by two authors (AS, PP). All except two authors (GL, PPo) assessed full texts for eligibility. Multiple reports from the same study were identified. Data from published reports was extracted using a prespecified data collection form. Data describing study and patient characteristics, number of patients in intervention and control arm, number and percent of patients with new AF, median CHA_2_DS_2_-VASc score, percentage of patients scoring ≥2 and data necessary to perform risk of bias assessment were extracted. Risk of bias assessment was performed by two authors (AS, PP). Disagreements were resolved by discussion with all other authors. Randomized controlled studies were assessed according to the Cochrane Collaboration’s tool and in prospective cohort studies the Newcastle-Ottawa Scale was adopted.

For any individual study a risk difference was calculated. In single-arm studies the risk in control group was assumed to be 0. An intention-to-treat analysis to estimate new AF incidence was conducted, the denominator being all patients qualified to (be screened) who were eligible and consented, even if differently assessed in original study reports.

The methodological (study design, quality) and clinical (population, setting, systematic vs. opportunistic screening) heterogeneity was assessed. It was decided to perform meta-analysis to estimate new AF incidence as a result of screening using data collected from all eligible studies and to address the heterogeneity in subgroup and sensitivity analysis. Statistical heterogeneity was evaluated with Cochran's Q-statistic and quantified with the I^2^ statistic. Whenever tests for heterogeneity revealed that variations between the studies were statistically significant (P < 0.05 or I^2^ ≥ 50%), a random-effects model was used. Subgroup analyses were carried out as follows: cut-off age: >64 vs. <65 years old, setting: GP vs. community, type of intervention: pulse palpation vs. ECG, 12-lead ECG vs. less than 12-lead ECG, systematic vs. opportunistic screening, heart rhythm measurement frequency: repeated vs. only once, randomized clinical trials vs. observational studies. In addition, five potential sources of heterogeneity: age, screening setting, ECG vs. pulse palpation, systematic vs. opportunistic screening and heart rhythm measurement frequency were tested by meta-regression in a multivariate meta-regression model. In sensitivity analysis studies in which recruited participants had to have one of the following: AF or stroke risk factor, heart disease (coronary heart disease, congestive heart failure) were excluded. Egger's test and Begg's funnel plot were used to evaluate the publication bias. All statistical analyses were performed using Statistica version 12.0 (StatSoft, Tulsa, US) and all tested P values < 0.05 were considered statistically significant.

## Results

**Fig 1:**
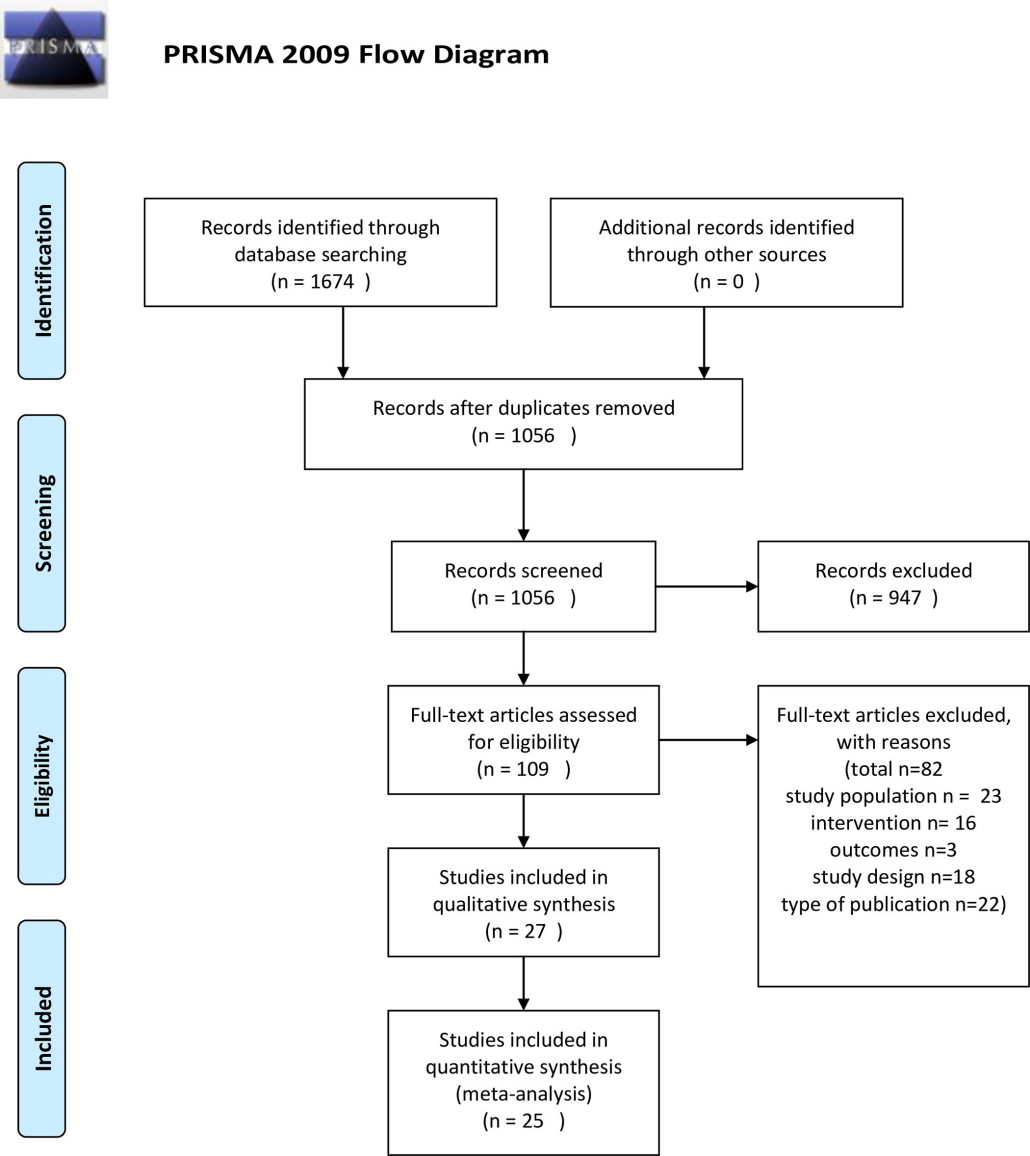
Details of the study selection. In total, 1056 articles were found. A primary screen of the abstracts resulted in the exclusion of 947 records. A further 82 were excluded based on the full-text review. This produced 27 articles that met inclusion criteria though representing 25 unique (3 RCTs and 22 observational) studies.

Details of the study selection are presented in Fig 1. Table 1 displays the characteristics and outcomes of included studies. 25 studies that were included came from 14 countries. Participants were recruited from GPs (10 studies) or community (15 studies). Concerning the study design, these included 3 randomized clinical trials [11–13], 4 prospective cohort studies with randomization [9,14–16], 13 prospective cohort studies [17–29], 4 cross-sectional studies [30–33], and one case-control study[34].The total number of participants was 88 786 with weighted mean age of 58.6 and 46.4% were males. The lower age limit for recruitment varied widely across studies with 13 studies limiting recruitment to > 64 years [9,11,12,14,15,17,22–24,28,30]. In 5 studies [13,18,21,25,26] recruited participants had to have one of the following: AF or stroke risk factor, coronary heart disease, congestive heart failure. The most common exclusion criteria were: atrial fibrillation or atrial flutter, prior stroke, transient ischemic attack, implantable pacemaker or defibrillator, terminal illness or severe cognitive impairment.

**Table 1:** Characteristics and outcomes of included studies.

Study	Country	Study design	Population cut-off age (mean); setting	Intervention	N	New AF (%)
Smyth 2015 [17]	Ireland	Prospective cohort	>64 years (75.1); GP	Pulse palpation, repeatedly, opportunistic	7262	55 (0.8)
Svennberg 2015 [9]	Sweden	Prospective cohort, randomisation	75–76 years (75.5); community	Less than 12-lead ECG, repeatedly, systematic	7173	218 (3.0)
Bury 2015 [14]	Ireland	Prospective cohort, randomisation	≥70 years (78); GP	Less than 12-lead ECG, only once, systematic	566	12 (1.2)
Turakhia 2015 [18]	USA	Prospective cohort	≥55 years (69); outpatient clinic	Less than 12-lead ECG (Zio wearable patch-based device), repeatedly, opportunistic	75	4 (5.3)
Benito 2015[13]	Spain	RCT	(69), primary healthcare centre	12-lead ECG, repeatedly, systematic (intervention group)	463	11 (2.4)
Lowres 2014 [30]	Australia	Cross-sectional	≥ 65 years (76); community	Less than 12-lead ECG, only once, opportunistic	1000	15 (1.5)
Javed 2014 [22]	UK	Prospective cohort	>65 years (69.7); GP	Less than 12-lead ECG, only once, systematic	6856	54 (0.8)
Van Mourik 2014 [23]	Netherlands	Prospective cohort	≥65 years (75.5); GP	12-lead ECG, only once, systematic	389	7 (1.8)
Virtanen 2014 [15]	Finland	Prospective cohort study, randomisation	≥75 years (79); community	Pulse palpation, repeatedly, systematic	205	4 (1.9)
Clua-Espuny 2013 [16]	Spain	Prospective cohort, randomisation	>60 years; GP	12-lead ECG, only once, systematic	1043	23 (2.2)
Rhys 2013 [24]	UK	Prospective cohort	≥65 years; community	Pulse palpation, only once, opportunistic	573	2 (0.3)
Hendrikx 2013 [25]	Sweden	Prospective cohort	(69.8); GP	Less than 12-lead ECG, repeatedly, opportunistic	928	35 (3.8)
Wiesel 2013 [26]	USA	Prospective cohort	>64 years or those with hypertension, diabetes, congestive heart failure, or previous stroke (67); GP	Less than 12-lead ECG (AF-BP monitor), repeatedly, systematic	139	2 (1.4)
Frewen 2013 [27]	Ireland	Prospective cohort	≥50 years (63.8); community	12-lead ECG, only once, systematic	4890	45 (0.9)
Sanmartin 2013 [28]	Spain	Prospective cohort	≥65 years; community	Pulse palpation, only once, systematic	1532	17 (1.1)
Claes 2012 [31]	Belgium	Cross-sectional	≥40 years (59); community	Less than 12-lead ECG, only once, opportunistic	10 758	167 (1.6)
Schnabel 2012[33]	Germany	Cross-sectional	(52.2); community	12-lead ECG, only once, systematic	5000	25 (0.5)
Doliwa 2009 [32]	Sweden	Cross-sectional	Community	Less than 12-lead ECG, only once, opportunistic	606	6 (1.0)
Yap 2008 [29]	Singapore	Prospective cohort	≥55 years; community	12-lead ECG, only once, systematic	1839	16 (0.9)
Kim 2007 [34]	Korea	Case-control	(49_median); community	12-lead ECG, repeatedly, opportunistic	16 568	61 (0.4)
Fitzmaurice 2007 [11]	UK	RCT	≥65 years (75.3); GP	12-lead ECG, only once, systematic	4562	74 (1.62)
				Pulse palpation, repeatedly, opportunistic	4575	75 (1.64)
Minami 2007 [19]	Japan	Prospective cohort	(51); community	12-lead ECG, only once, opportunistic	722	5 (0.7)
Scalvini 2005 [20]	Italy	Prospective cohort	(61); GP	12-lead ECG, repeatedly, systematic	7516	271 (3.6)
Rockman 2004 [21]	USA	Prospective cohort	>60 years (70.8); community	12-lead ECG, only once, opportunistic	610	3 (0.5)
Morgan 2002 [12]	UK	RCT	65 to 100 years; GP	Pulse palpation, only once, systematic	1499	39 (2.6)
				Pulse palpation, only once, opportunistic	1437	15 (1.0)

AF–atrial fibrillation, AF-BP–atrial fibrillation-blood pressure, ECG–electrocardiogram, GP–general practitioner, RCT–randomised clinical trial

In 14 studies [9,11–16,20,23,26–29,33] screening was conducted systematically, whereas in 13 studies [11,12,17–19,21,22,24,25,30–32,34] it was done opportunistically. In the SAFE study [11] there were two screening arms: systematic and opportunistic. Similarly, in the RCT *Morgan 2002* [12] systematic and opportunistic groups were compared. These were analysed separately for the calculation of main outcome in subgroup analysis. Screening modalities differed between studies: in 11 [11,13,16,19–21,23,27,29,33,34] it was a 12-lead ECG; in 9 [9,14,18,22,25,26,30–32] less than 12-lead (1-3-lead) ECG; and in 7 [11,12,15,17,24,28] pulse palpation, usually followed by ECG if the pulse was irregular. In 17 studies [11,12,14,16,19,21–24,27–33] there was only one heart rhythm measurement performed, while in 10 [9,11,13,15,17,18,20,25,26,34] studies the rhythm was taken several times within a certain time period.

### Risk of bias in included studies

Overall quality of non-randomized studies was moderate, according to the Newcastle-Ottawa Scale. In four studies [18,21,25,26] the population was not representative of the average population. In one study [15] it was not possible to objectively confirm whether screening actually took place. In two studies [27,34] it was possible to include patients with already known AF in the main outcome assessment. In three studies [18,31,32]the diagnosis of AF was not confirmed in an objective or blinded fashion, i.e. by the 12-lead ECG interpreted independently by a cardiologist. Only in eight studies [9,15,17,18,20,25,26,34], in which heart rhythm measurements were being repeated, follow-up was considered long enough for outcome to occur. In one study [23] the proportion of patients eligible for and agreeing to screening who actually were screened did not exceed 80%. In two randomised trials [11,12] risk of bias assessed according to the Cochrane Collaboration’s tool was relatively low and in one[13]–relatively high. The details of risk of bias assessment are presented in the Tables A-D in S2 Appendix.

### Effects of interventions

#### Newly identified AF

**Fig 2:**
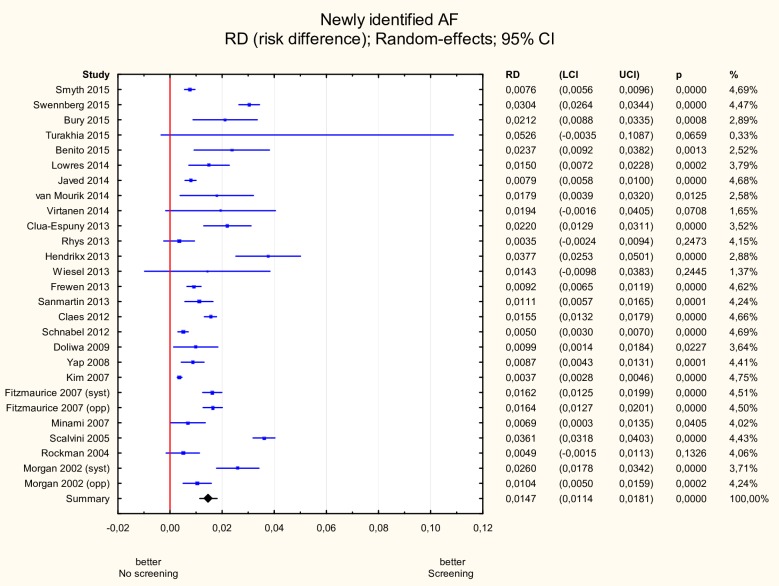
Meta-analysis of new AF incidence due to screening. Three randomized trials and 22 observational studies were included. The total number of participants was 88 786. Heterogeneity was high at I2 = 95.13% (Q = 534, p<0.05). The incidence of newly detected AF was 1.5% (95% CI 1.1–1.8%). AF—atrial fibrillation.

The incidence of newly detected AF was 1.5% (95% CI 1.1–1.8%) (Fig 2). The hypothesis about the homogeneity of the studies was rejected therefore a random effects model was implemented (Q = 534, p<0.05, I^2^ = 95.13%).

#### Age cut-off for screening

When screening was limited to participants from studies with entry cut-off ≥65 years (mean age 67.2 years), the incidence was 1.5% (95% CI 1.0–2.0%) as compared to 1.4% (95% CI 1.0–1.9%) among participants from studies where entry cut-off was 40–64 years (mean age 52.2 years). This difference was not statistically significant (Table 2).

**Table 2:** Results of subgroup meta-analysis.

Comparison	Group 1	Number of studies	Number of participants	% AF identified	Group 2	Number of studies	Number of participants	% AF identified	p
Age	>64	13	37 629	1.5 (1.0–2.0)	<65	14	51 157	1.4 (1.0–1.9)	>0.05
Setting	GPs	12	35 273	1.9 (1.4–2.4)	Community	15	53 513	1.1 (0.7–1.6)	<0.05
ECG vs. pulse palpation	ECG	20	71 703	1.6 (1.2–2.0)	Pulse palpation	7	17 083	1.3 (0.6–1.9)	>0.05
12-lead ECG vs. less than 12-lead ECG	12-lead ECG	11	43 602	1.3 (0.8–1.9)	Less than 12-lead ECG	9	28 101	1.9 (1.2–2.6)	>0.05
Systematic vs. opportunistic	Systematic	14	36 816	1.8 (1.4–2.3)	Opportunistic	13	51 970	1.1 (0.6–1.6)	<0.05
Method of screening	Pulse palpation systematic	3	3 236	1.8 (0.7–3.0)	Pulse palpation opportunistic	4	13 847	1.0 (01–1.8)	>0.05
	ECG systematic	11	33 580	1.8 (1.3–2.4)	ECG opportunistic	9	38 123	1.2 (0.6–1.8)	
HR measurement frequency	Repeated	10	44 904	2.1 (1.5–2.8)	Only once	17	43 882	1.2 (0.8–1.6)	<0.05

AF–atrial fibrillation, ECG–electrocardiogram, GP–general practitioner

#### Stroke risk

CHA_2_DS_2_-VASc score, which describes the risk of thromboembolic complications, was reported in 11 studies [9,13,14,16–18,24,27,30,31,33], in one study [25] CHADS_2_ score was calculated. In 9 studies [9,13,14,17,18,24,25,30,33] the stroke risk for previously unknown AF was reported separately. In all 9 studies, median CHA_2_DS_2_-VASc score or CHADS_2_ score was not lower than 2. A vast majority of patients with newly detected AF (90.9–100%) scored ≥2, which made them eligible for OACs.

#### Screening setting

**Fig 3:**
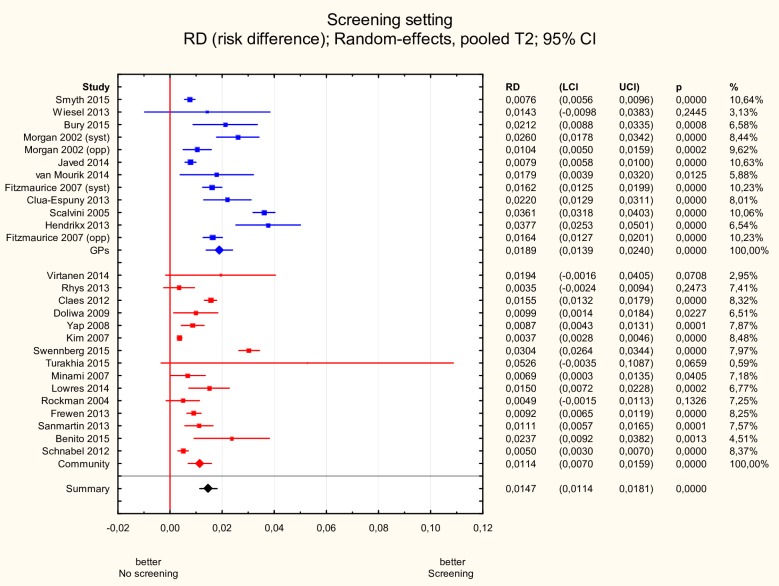
Subgroup meta-analysis of new AF incidence according to setting. New AF incidence was significantly higher in studies (n = 12) in which screening was performed in GP setting compared to studies in which screening was done in the community (n = 15): 1.9% (95% CI 1.4–2.4%) vs. 1.1% (95% CI 0.7–1.6%), p<0.05. AF—atrial fibrillation.

AF incidence was significantly higher in the GP setting than the community setting: 1.9% (95% CI 1.4–2.4%) vs. 1.1% (95% CI 0.7–1.6%), p<0.05 (Fig 3).

#### ECG vs. pulse palpation

Different screening techniques were compared one to each other and no statistical differences were found whether pulse palpation or ECG was used as a primary screening modality (1.3% vs. 1.6%, respectively). 12-lead ECG was numerically worse compared to strategies other than 12-lead ECG in identifying new AF cases, but this difference again was not significant: 1.3% vs. 1.9%.

#### Systematic vs. opportunistic screening

**Fig 4:**
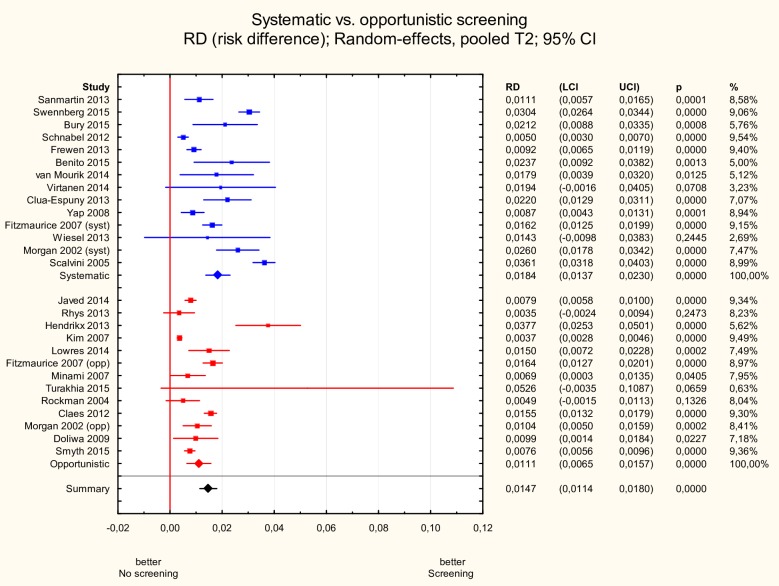
Subgroup meta-analysis of new AF incidence according to systematic vs. opportunistic screening. New AF incidence was significantly higher in studies (n = 14) in which screening was organized in a systematic way in contrast to studies where it was opportunistic (n = 13): 1.8% (95% CI 1.4–2.3%) vs. 1.1% (95% CI 0.6–1.6%), p<0.05. AF—atrial fibrillation.

The incidence of newly detected AF was significantly higher in studies in which screening was organized in a systematic way in contrast to studies where it was opportunistic: 1.8% (95% CI 1.4–2.3%) vs. 1.1% (95% CI 0.6–1.6%), p<0.05 (Fig 4).

When studies were subdivided in relation to screening technique and screening organizational process, the differences, though not significant, tended to be greater between systematic screening vs. opportunistic screening groups than between pulse palpation-based vs. ECG-based groups (Table 2).

#### Single vs. repeated measurements

**Fig 5:**
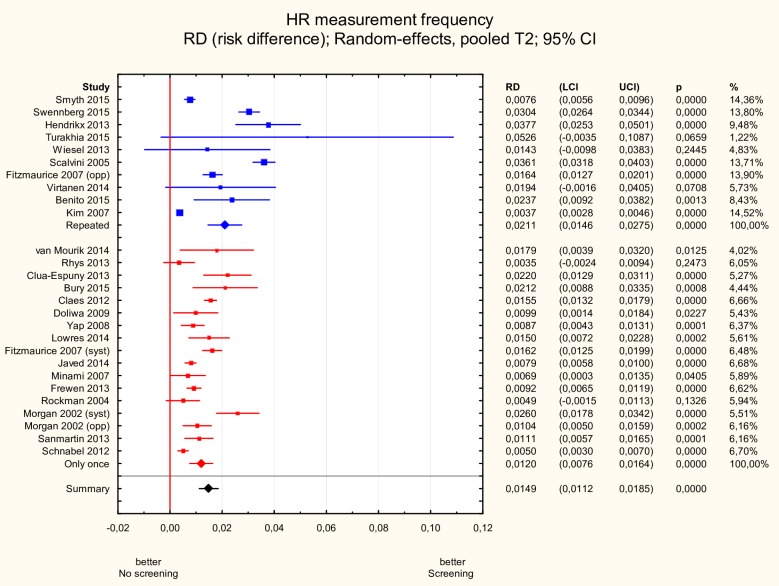
Subgroup meta-analysis of new AF incidence according to single vs. repeated screening. New AF incidence was significantly higher in studies (n = 10) in which heart rhythm was measured repeatedly as opposed to studies with a single measurement performed (n = 17):: 2.1% (95% CI 1.5–2.8%) vs. 1.2% (95% CI 0.8–1.6%), p<0.05. AF—atrial fibrillation.

Studies in which heart rhythm was measured repeatedly within a certain time period as opposed to a single measurement reported significantly higher new AF incidence: 2.1% (95% CI 1.5–2.8%) vs. 1.2% (95% CI 0.8–1.6%), p<0.05 (Fig 5).

#### Study design

The incidence of newly identified AF was not different in RCTs in comparison with observational studies: 1.8% (95% CI 1.0–2.5%) vs. 1.4% (95% CI 1.0–1.8%), p>0.05.

### Sensitivity analysis

In sensitivity analysis we excluded studies in which recruited participants had to have either of the following: AF or stroke risk factor, coronary heart disease, congestive heart failure [13,18,21,25,26]. However, the incidence of new AF was grossly unchanged and equalled 1.4% (95% CI 1.0–1.8%).

### Meta-regression analysis

Results of the meta-regression analysis are presented in Table 3. Only heart rhythm measurement frequency had statistical significance in the multivariate model (p<0.05).

**Table 3:** Results of meta-regression analysis.

Variable	Regression coefficient	Standard error	Lower 95% CI	Upper 95% CI	p
Systematic screening (ref. opportunistic)	0.0064	0.0039	-0.0013	0.0141	0.103
ECG (ref. pulse palpation)	0.0052	0.0056	-0.0059	0.0162	0.360
Age >64 (ref. <65)	0.0013	0.0052	-0.0089	0.0115	0.803
Repeated HR measurement (ref. single)	0.0089	0.0043	0.0005	0.0173	0.037
GP (ref. community)	0.0062	0.0043	-0.0022	0.0147	0.146

### Publication bias

**Fig 6:**
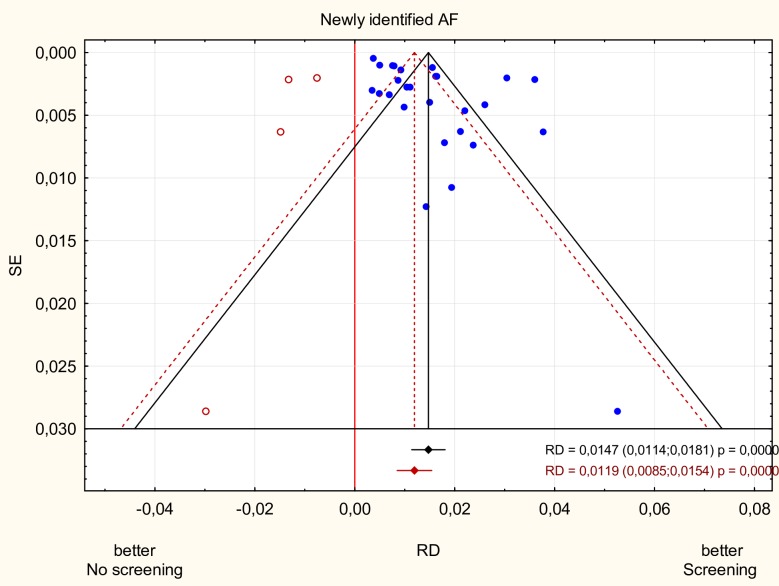
Funnel plot of newly identified AF. Begg’s funnel plot of newly identified AF was asymmetric suggesting the presence of publication bias. Open circles represent the imputed sudies to adjust the analysis for the effect of potential publication bias. AF—atrial fibrillation.

Asymmetric Begg’s funnel plot of newly identified AF together with the result of Egger's test (p = 0.002) suggested the presence of publication bias. Using ‘‘trim and fill” method, four potentially missing studies were imputed on the left side of the funnel plot, yielding an adjusted risk difference of 1.2% (95% CI 0.8–1.5%) (Fig 6).

## Discussion

In this systematic review and meta-analysis of contemporary studies, our principal findings are as follows: (i) active screening for previously undiagnosed AF is more effective than no screening in terms of detection of new cases of AF in various populations of patients above 40 years of age; (ii) the incidence of “silent” AF in the current study was 1.5% which is consistent with the previous report [35]; and (iii), the organisation of screening process may be more important than technical solutions used for heart rhythm assessment.

In the present meta-analysis we analysed results of 25 studies employing different screening modalities and approaches. This allowed for the comparison of various aspects of the screening process. Such assessment was not possible in earlier review by Moran et al.[36] due to strict inclusion criteria resulting in analysis of only one randomized controlled trial. Two recent systematic reviews differed from our work due to higher age cut-off (≥65 years) for patients included in analysed studies or exclusion of non-randomized studies. Nonetheless, both reviews reported superiority of screening comparing to no screening for detection of previously undiagnosed AF[37,38].

Superiority of the systematic approach in identifying previously undiagnosed AF was shown in the subgroup analysis of the current meta-analysis. Interestingly, higher efficiency of systematic vs. opportunistic screening did not depend on the age-cut off in meta-regression analysis. It suggests that systematic screening programmes may also be effective when addressed towards individuals younger than septuagenarians and octogenarians.

When comparing systematic and opportunistic approach it should be noted that the results of SAFE trial questioned the cost-effectiveness of the systematic screening [11,39] performed in broad group of patients age ≥ 65 years. However, the analysis of STROKESTOP study showed that if carried out in precisely targeted population (75 to 76-years-old) the systematic approach may well be cost-effective[40].

In our analysis, we also divided analysed studies into two groups based on the age cut-off: (1) below 65 years and (2) equal or above 65 years. This particular cut-off was chosen in accordance to ESC guidelines[8]–recommendation based on the entry criteria and the results of SAFE study[11,39]. We found no difference in terms of the effectiveness of screening aimed at patients ≥ 65 in comparison to screening employing lower cut-offs of age. However, it must be noted that actual mean age of participants in these two groups of studies while undoubtedly different (52.2 vs. 67.2 years) was still relatively low. Furthermore, while shifting down the cut-off age for the participation in screening programmes to values between 40 and 65 years might result in a similar detection rate of new AF (and thus higher absolute number of new cases), it might not necessarily be a cost-effective approach as significant proportion of patients below 65 years will not require oral anticoagulation due to low CHA_2_DS_2_-VASc scores[8]. This aspect was not assessed in our analysis.

Of interest, we showed that GP-led screening (carried out in local practices by primary care physicians) was more effective than screening performed in the community. This observation was proven to be valid also in meta-regression analysis which suggests its independence from other aspects of screening. Similar findings were reported in previous systematic review by Lowres et al.[35] It remains to be found which characteristics of GP-led screening give this strategy an edge over community based screening. Among them the specific features of individuals attending GP surgeries (e.g. concomitant diseases) might increase the detection rate of previously unknown AF[41]. Regardless of the potential explanation, our results clearly suggest that the GP-led approach might be a preferable method for further screening programmes when compared to the community based initiatives.

Importantly, the majority of patients (90.9% to 100%) with previously unknown AF qualified for the anticoagulation treatment because of high thromboembolic risk[8]. Introduction of such therapy might be of particular clinical [4,5] and economic benefit [40] for these individuals and thus further reinforces the practice of active screening.

Also, the current meta-analysis shows a similar effectiveness of screening modalities based on ECG comparing to pulse palpation when used as initial assessment of the cardiac rhythm. While incidence of undiagnosed AF was slightly higher with ECG (1.6% vs. 1.3%), the difference was not statistically meaningful. It is a reassuring finding as pulse palpation, when performed by appropriately trained personnel, is more widely available as a first step of screening for AF than ECG. Additionally, we confirmed non-inferiority of ECG systems using less than 12-leads tracings comparing to standard 12-lead ECG. In fact, the incidence of newly detected AF was numerically greater in studies employing simplified ECG recordings.

Carrying out the repeated testing increased the detection rate of new AF and in fact, heart rhythm measurement frequency was the only variable that showed statistical significance in a multivariate meta-regression model. It is worth to note that the review by Lowres et al.[35] included studies that screened on only one occasion. Interestingly in STROKESTOP study intermittent monitoring diagnosed 4 times as many individuals with new AF compared with the initial ECG[9]. Patients with permanent AF may have already been diagnosed, whereas paroxysmal AF is more problematic to detect with single time-point measurement, as patients may be in sinus rhythm when screened. User-friendly features of less than 12-lead ECG systems make the simplified and repeatable ECG approaches a viable option for further screening programmes for “silent” AF–especially in individuals with low-burden, paroxysmal AF.

In contrast to ESC guidelines the UK National Screening Committee (NSC) does not advocate population screening for AF[42] based on: (1) lack of high-quality evidence coming from randomised controlled trials confirming that screening for silent AF saves lives or reduces morbidity and (2) uncertainty whether risk of stroke in someone with screen-detected AF is the same as in individual with AF detected due to clinical presentation. However, in large UK cohort study[43] it was shown that patents with incidentally detected ambulatory AF are characterized by high risk of stroke which can be significantly reduced (similarly as the risk of death) by introduction of anticoagulation treatment as compared to no therapy. Thus, the British Cardiovascular Society issued a subsequent statement[44], in response to rather conservative recommendation of NSC, suggesting that it would be in the public interest to reconsider their decision. We believe that results of our study (among other recent publications[37,38]) support the concept of wide screening for AF.

### Limitations

Our study is not without limitations. First of all, the heterogeneity of analysed studies must be appreciated. Secondly, only three studies[11–13] were performed in a randomized manner and only these included control groups what may question the reliability of the results obtained. In fact, a proportion of new AF diagnoses may be due to incidental findings or development of symptoms within the screening period. Finally, we analysed the effectiveness of different aspects of the screening process only in relation to new cases of AF. Therefore, we were not able to assess the incidence of patients with previously diagnosed AF who were not using adequate anticoagulation. Recognition of such individuals may be clinically as important as finding patients with newly identified AF.

## Conclusions

In conclusion, this meta-analysis showed that active screening for undiagnosed AF is efficacious beginning from 40 years of age. Our results suggest that the organisation of screening process (GP-led, systematic and repeated screening) is more important than technical solutions used for the heart rhythm assessment.

## Supporting information

S1 Table PRISMA checklist. (DOC)

S1 Appendix Search strategies for EMBASE and MEDLINE. (DOCX)

S2 Appendix (Tables A-D). Risk of bias assessment.
(DOCX)
