# Supplementary material for: Effectiveness of screening for atrial fibrillation and its determinants. A meta-analysis
Source: PLoS One. 2019 Mar 20;14(3):e0213198. doi: 10.1371/journal.pone.0213198 (PMC6426211; doi:10.1371/journal.pone.0213198)
Supplement: S1 Appendix — (DOCX) [file pone.0213198.s002.docx]

Search strategies for EMBASE and MEDLINE

A: EMBASE

(('atrial fibrillation':de OR 'atrial fibrillation':ab,ti) AND [embase]/lim AND [2000-2015]/py) AND (('mass screening':de OR 'mass screening':ab,ti OR 'screening':ab,ti OR 'detection':ab,ti OR 'case finding':ab,ti) AND [embase]/lim AND [2000-2015]/py) AND (('pulse rate':de OR 'pulse rate':ab,ti OR 'electrocardiogram':de OR 'electrocardiogram':ab,ti OR 'electrocardiography':ab,ti OR 'ecg':ab,ti) AND [embase]/lim AND [2000-2015]/py)

|  |  | Embase |
| --- | --- | --- |
| 1 | 'atrial fibrillation':de OR 'atrial fibrillation':ab,ti | 86,625 |
| 2 | 'mass screening':de OR 'mass screening':ab,ti OR 'screening':ab,ti OR 'detection':ab,ti OR 'case finding':ab,ti | 749,490 |
| 3 | 'pulse rate':de OR 'pulse rate':ab,ti OR 'electrocardiogram':de OR 'electrocardiogram':ab,ti OR 'electrocardiography':ab,ti OR 'ecg':ab,ti | 123,015 |
| 4 | #1 AND #2 AND #3 | 1026 |

B: MEDLINE (PubMed)

(((((Atrial fibrillation[MeSH Terms]) OR Atrial fibrillation[Title/Abstract]) AND ("2000/01/01"[Date - Publication] : "2015/12/31"[Date - Publication]))) AND ((((((Mass screening[MeSH Terms]) OR Mass screening[Title/Abstract]) OR Screening[Title/Abstract]) OR Detection[Title/Abstract]) OR Case finding[Title/Abstract]) AND ("2000/01/01"[Date - Publication] : "2015/12/31"[Date - Publication]))) AND (((((((Pulse[MeSH Terms]) OR Pulse[Title/Abstract]) OR Electrocardiography[MeSH Terms]) OR Electrocardiography[Title/Abstract]) OR Electrocardiogram[Title/Abstract]) OR ECG[Title/Abstract]) AND ("2000/01/01"[Date - Publication] : "2015/12/31"[Date - Publication]))

|  |  |  |
| --- | --- | --- |
| 1 | Atrial fibrillation [MeSH] |  |
| 2 | Atrial fibrillation [ta] |  |
| 3 | #1OR#2 | 42 412 |
| 4 | Mass screening [MeSH] |  |
| 5 | Mass screening [ta] |  |
| 6 | Screening [ta] |  |
| 7 | Detection [ta] |  |
| 8 | Case finding [ta] |  |
| 9 | #4OR#5OR#6OR#7OR#8 | 709 951 |
| 10 | Electrocardiography [MeSH] |  |
| 11 | Electrocardiography [ta] |  |
| 12 | ECG [ta] |  |
| 13 | Electrocardiogram [ta] |  |
| 14 | Pulse [MeSH] |  |
| 15 | Pulse [ta] |  |
| 16 | #10OR#11OR#12OR#13OR#14OR#15 | 207 249 |
| 17 | #3AND#9AND#16 | 648 |
