# Supplementary material for: Effectiveness of screening for atrial fibrillation and its determinants. A meta-analysis
Source: PLoS One. 2019 Mar 20;14(3):e0213198. doi: 10.1371/journal.pone.0213198 (PMC6426211; doi:10.1371/journal.pone.0213198)
Supplement: S2 Appendix — (Tables A-D). Risk of bias assessment. (DOCX) [file pone.0213198.s003.docx]

Table A. Risk of bias assessment of non-randomized studies (n=21) according to the Newcastle-Ottawa Scale.

| STUDY | SELECTION | | | | COMPARABILITY | OUTCOME | | |
| --- | --- | --- | --- | --- | --- | --- | --- | --- |
|  | S1 | S2 | S3 | S4 | n/a | O1 | O2 | O3 |
| Smyth 2015 | * | n/a | * | * |  | * | * | * |
| Svennberg 2015 | * |  | * | * |  | * | * | * |
| Bury 2015 | * |  | * | * |  | * | - | * |
| Turakhia 2015 | - |  | * | * |  | - | * | * |
| Lowres 2014 (187) | * |  | * | * |  | * | - | * |
| Javed 2014 | * |  | * | * |  | * | - | * |
| Van Mourik 2014 | * |  | * | * |  | * | - | - |
| Virtanen 2014 | * |  | - | * |  | * | * | * |
| Clua-Espuny 2013 | * |  | * | * |  | * | - | * |
| Rhys 2013 (400) | * |  | * | * |  | * | - | * |
| Hendrikx 2013 (419) | - |  | * | * |  | * | * | * |
| Wiesel 2013 (420) | - |  | * | * |  | * | * | * |
| Frewen 2013 | * |  | * | - |  | * | - | * |
| Sanmartin 2013 | * |  | * | * |  | * | - | * |
| Claes 2012 | * |  | * | * |  | - | - | * |
| Schabel 2012 | * |  | * | * |  | * | - | * |
| Doliwa 2009 | * |  | * | * |  | - | - | * |
| Yap 2008 | * |  | * | * |  | * | - | * |
| Kim 2007 | * |  | * | - |  | * | * | * |
| Minami 2007 | * |  | * | * |  | * | - | * |
| Scalvini 2005 | * |  | * | * |  | * | * | * |
| Rockman 2004 | - |  | * | * |  | * | - | * |

Table B. Risk of bias assessment of *Benito 2015* according to the Cochrane Collaboration’s tool.

| **Bias** | **Author’s judgement** | **Support for judgement** |
| --- | --- | --- |
| Random sequence generation (selection bias) | Unclear risk | Quote: A simple random sample of 4000 candidates was preselected from the  reference population. Patients were then randomized, with a 1:1 allocation  ratio, and consecutively assigned to either the intervention or control group.  Comment: Probably done. Although authors stated that patients were randomized, method of randomization was not fully described. |
| Allocation concealment (selection bias) | Low risk | Quote: Patients were then randomized, with a 1:1 allocation  ratio, and consecutively assigned to either the intervention group  (IG) or the control group (CG), until the required sample was achieved.  Comment: Probably done.  Authors stated that patients were consecutively assigned to groups. |
| Blinding of participants and personnel (performance bias) | High risk | Quote: -  Comment: Probably not done.  Blinding was impossible due to the fact that in the intervention group ECG was performed, while in control group no specific action was taken. |
| Blinding of outcome assessment (detection bias) | High risk | Quote: -  Comment: Probably not done.  Blinding of outcome assessment was impossible due to the fact that in control group ECG was not performed. |
| Incomplete outcome data (attrition bias) | Low risk | Effect sizes were estimated on an intention to treat basis.  Comment: Probably done. |
| Selective reporting (reporting bias) | Low risk | Quote:-  Comment. Probably done. Study protocol not available, though outcomes of all endpoints listed in the publication were presented. |
| Other bias | Unclear risk |  |

Table C. Risk of bias assessment of *Fitzmaurice 2007* according to the Cochrane Collaboration’s tool.

| **Bias** | **Author’s judgement** | **Support for judgement** |
| --- | --- | --- |
| Random sequence generation (selection bias) | Low risk | Quote: After stratification for practice size and deprivation we used MINITAB to select randomly two equal size groups from those practices within a particular stratum (main trial). After stratification for known atrial fibrillation we randomly allocated patients from the 25 intervention practices to systematic or opportunistic screening. We obtained a computerised list of all patients aged 65 or over from each practice. Random selection of patients for each practice was achieved with computer generated random numbers (MINITAB) to access entries in lists of patients. We used SPSS to allocate patients randomly from this list to either systematic or opportunistic screening to create two equal size groups of patients within each stratum so that each strategy (systematic or opportunistic screening) had an equal chance of detecting known, unknown, and suspected atrial fibrillation. We used a simulated value from a Bernoulli distribution, comprising two values equally likely to occur, to determine which group became the intervention arm and then the systematic arm.  Comment: Probably done. |
| Allocation concealment (selection bias) | Unclear risk | Quote: There was no deliberate concealment of allocation to the trial arms.  Comment: Probably not done. |
| Blinding of participants and personnel (performance bias) | Unclear risk | Quote: Practice nurses ran the screening clinics and collected baseline information and medical history (including any previous diagnosis of atrial fibrillation), took the radial pulse, and carried out 12 lead electrocardiography.  Comment: Probably not done. However, practice nurses were not aware of the prior diagnosis of AF. |
| Blinding of outcome assessment (detection bias) | Low risk | Quote: Two consultant cardiologists, who were blinded to allocation, assessed whether the electrocardiogram showed atrial fibrillation or not and identified any other relevant abnormalities. A third blinded cardiologist arbitrated on any disagreements over diagnosis.  Comment: Probably done. |
| Incomplete outcome data (attrition bias) | Low risk | 1) Electrocardiography performed (n=2357)/4933 patients allocated to total population systematic arm  2) Pulses recorded (n=3278)/4933 patients allocated to opportunistic screening arm  Comment: Probably done. Authors analyzed data on an intention to treat basis. |
| Selective reporting (reporting bias) | Low risk | Comment. Probably done. All outcomes specified in the trial protocol were reported. |
| Other bias | Unclear risk |  |

Table D. Risk of bias assessment of *Morgan 2002* according to the Cochrane Collaboration’s tool.

| **Bias** | **Author’s judgement** | **Support for judgement** |
| --- | --- | --- |
| Random sequence generation (selection bias) | Low risk | Quote: Approximately 750 patients from each practice list were randomly selected to give a total study sample of 3001. These patients were randomised to receive either an invitation to nurse-led systematic screening, or to opportunistic screening prompted by a reminder flag placed in their medical records.  Comment: Probably done. |
| Allocation concealment (selection bias) | Unclear risk | Quote: Patients randomised to systematic screening were sent by post an invitation to attend a specific appointment at their own general practice to see a nurse. Patients in the opportunistic screening arm had the reminder flag placed in the notes for a six-month period. Any doctor or nurse who made an assessment of the pulse was asked to indicate...  Comment: Probaby not done. It was impossible to conceal allocation from the personnel. |
| Blinding of participants and personnel (performance bias) | Unclear risk | Quote: as above  Comment: Probably not done. |
| Blinding of outcome assessment (detection bias) | Low risk | Quote: The ECG’s rhythm strips were read centrally.  Comment: Probably done. |
| Incomplete outcome data (attrition bias) | Low risk | Uptake in the systematic screening arm was 73%,compared to 29% in the opportunistic arm. However, effect sizes were estimated on an intention to treat basis.  Comment: Probably done. |
| Selective reporting (reporting bias) | Low risk | Comment. Probably done. All outcomes specified in the trial protocol were reported. |
| Other bias | Unclear risk |  |
